# Grain yield and quality responses of wheat expressing a barley sucrose transporter to combined climate change factors

**DOI:** 10.1093/jxb/erx366

**Published:** 2017-10-21

**Authors:** Heiko Weichert, Petra Högy, Isabel Mora-Ramirez, Jörg Fuchs, Kai Eggert, Peter Koehler, Winfriede Weschke, Andreas Fangmeier, Hans Weber

**Affiliations:** 1 Leibniz Institut für Pflanzengenetik und Kulturpflanzenforschung, D-06466 Gatersleben, Germany; 2 University of Hohenheim, Institute of Landscape and Plant Ecology, Department of Plant Ecology and Ecotoxicology, D-70599 Stuttgart, Germany; 3 Deutsche Forschungsanstalt für Lebensmittelchemie; Leibniz Institut, Lise-Meitner-Straße 34, D-85353 Freising, Germany

**Keywords:** Climate change, CO_2_ enrichment, grain quality, storage proteins, sucrose transport, wheat yield

## Abstract

Crop yield stability must be ensured under future climate conditions such as elevated CO_2_ and high temperatures. We tested ‘HOSUT’, a winter wheat line expressing a grain-targeted sucrose transporter of barley in response to combinations of CO_2_ enrichment, a heat wave, and high nitrogen fertilization. Compared with wild-type Certo, HOSUT had a superior performance for grain yield, aboveground biomass, and ears per plant, obviously due to transgene activity in developing grains and young vegetative sinks. HOSUT grains were larger and contained more endosperm cells. HOSUT and high CO_2_ effects similarly improved phenological and yield-related traits. Significant HOSUT–CO_2_ interactions for biomass of stems, ears, grain yield, nitrogen yield, and grain number revealed that Certo was promoted by CO_2_ enrichment, whereas HOSUT responded weakly. CO_2_ enrichment strongly reduced and HOSUT effects weakly reduced grain nitrogen, storage proteins, and free amino acids. In contrast to CO_2_ enrichment, HOSUT effects did not impair grain micronutrient concentrations. Significant HOSUT–nitrogen fertilization interactions for ear biomass, grain yield, grain number per plant, and harvest index indicated that HOSUT benefited more from additional nitrogen. The heat wave decreased aboveground and ear biomass, grain yield, harvest index, grain size, and starch and water use, but increased grain sucrose concentration.

## Teaser

HOSUT wheat, enhanced in grain sucrose transport, has a superior performance for yield-related traits, partially phenocopies effects from CO_2_ enrichment, and benefits more from high N fertilization.

## Introduction

Wheat accounts for 30% of global grain production and for 45% of cereal food, and therefore represents the major food plant. Grain filling depends on assimilate supply. Increased assimilate partitioning to developing spikes and grains had the greatest impact on improving yield potential in wheat in the past (Foulkes *et al.*, 2011). Grain yield is predominantly sink limited and grains grow under saturated source supply (Borrás *et al.*, 2004). Thus, improved assimilate uptake capacity and partitioning towards the grains can improve yield potential in wheat. Due to anthropogenic activities, the atmospheric carbon dioxide (CO_2_) concentration is projected to increase to 550 ppm by 2050, accompanied by higher global temperatures and increasing extreme weather events such as heat waves (Barriopedro *et al.*, 2011). Many studies also report observed increases in frequency, intensity, and/or longevity of heat waves, which can strongly impact plant growth (De Boeck *et al.*, 2010). While CO_2_ enrichment frequently improves carbon (C) assimilation and increases plant biomass in many species (Taub *et al.*, 2008), sink limitation often occurs, leading to photosynthetic feed-back inhibition (White *et al.*, 2016). This suggests that photosynthesis is only optimal at non-limiting sink capacity and therefore insufficient assimilate translocation could be critical (Aranjuelo *et al.*, 2011; Wang *et al.*, 2013).

CO_2_ enrichment causes reduced nitrogen (N) and protein levels in non-leguminous C_3_ species and alters acquisition, remobilization, redistribution, and concentration of N (Taub and Wang, 2008). CO_2_ enrichment physiologically induces N deficiency, reducing both N uptake from soil and N reduction from nitrate into amino N (Bloom *et al.*, 2014). Reduced N concentrations are only partially alleviated by increased N fertilization (Fangmeier *et al.*, 1999). Similarly, micronutrients such as S, Mg, Ca, and Fe, as well as Zn, Mn, and Cd are markedly reduced upon CO_2_ enrichment in wheat grains (Högy and Fangmeier, 2008; Pleijel and Högy, 2015). Thus, CO_2_ stimulation of grain yield is negatively correlated with the N status and may partially respond upon sufficient N supply (Kant *et al.*, 2012).

Elevated CO_2_ is the major anthropogenic greenhouse gas, and rising CO_2_ concentrations will influence growth and yield of crop plants via both its direct effects as substrate for photosynthesis and its indirect climatic effects. While future CO_2_ trends will probably increase global yields by ~1.8% per decade, warming trends are likely to reduce global yields by ~1.5% per decade (Lobell and Gourdji, 2012).

Grains take up sucrose and amino acids from the phloem (Weber *et al.*, 2005). Sucrose is important in transport and as a nutrient sugar, and also as a signal (Koch, 2004; Braun *et al.*, 2014; Griffiths *et al.*, 2016). Sucrose initiates seed maturation, signals the transition into the storage mode, thereby acting at transcriptional, hormonal, and metabolic levels, and is thus a key player within the regulatory network controlling seed maturation. Altering gene expression of assimilate transporters is promising for manipulating uptake capacity and partitioning. Expressing the amino acid permease gene *VfAAP1* in pea embryos increases amino acid supply, total seed N, and protein content, but leads to deregulated C to N balances (Weigelt *et al.*, 2008). Sucrose transporter overexpression in pea cotyledons also stimulates seed protein production (Rosche *et al.*, 2002, 2005).

The barley sucrose transporter gene *HvSUT1* has been ectopically expressed in winter wheat cv. Certo controlled by the barley *Hordein B1* promoter (HOSUT lines). We have shown earlier that in these lines sucrose uptake capacity and storage protein synthesis are stimulated (Weichert *et al.*, 2010). Repeated experiments in small unregulated greenhouses in field soil and with 400 plants m^–2^ revealed a large yield advantage for three independent HOSUT lines (Saalbach *et al.*, 2014). Higher thousand grain weight (TGW), grain width, and length pointed to increased individual grain sink strength and improved sucrose partitioning at the whole-plant level. The observed increase in individual grain size was somewhat compensated by lower grain numbers per spike. In addition, there was a trend towards lower grain protein concentrations. Finally, it was concluded that increased seed size and ear number per plant mostly contributed to the higher grain yield (Saalbach *et al.*, 2014).

The HOSUT lines are suitable models to better understand determinants of grain size and number, its relationship to grain sink strength, and the potential limitations of wheat yield potential. This study is based on existing data on three different wheat lines ectopically expressing a sucrose transporter. While yield advantages of these lines compared with the wild type have already been reported (Saalbach *et al.*, 2014), the lines have never been grown under varying assimilate source strength. CO_2_ enrichment means a potential increase in assimilate source strength both via increasing non-structural carbohydrate pools in the stems to be mobilized during grain filling and by stimulating flag leaf photosynthesis. At the same time, indirect effects of CO_2_ enrichment such as extreme climatic events may counteract this CO_2_ fertilization effect. As a third variable, N supply has to be taken into account since high N supply may possibly alleviate the trade-off between grain yield and grain protein concentration. Here, we wanted to analyse the combinatorial effects and interactions of elevated CO_2_, a heat wave applied during grain filling, and variation in N fertilization on growth and yield-related parameters of the HOSUT line as compared with the non-transgenic line Certo. The aim of this work was to test the hypothesis that the HOSUT line with improved sink strength can benefit more from source stimulation by CO_2_ enrichment compared with the wild-type control. Data on plant phenology, yield-related traits, grain dimensions, and composition were collected and investigated using a full four-factorial analysis.

## Materials and methods

### Experimental design and data analysis

A full factorial experiment was performed under controlled conditions in phytochambers with four factors, each with two levels. The factors were: (i) winter wheat (*Triticum aestivum* L. cv. Certo) HOSUT24 transgenic line (‘HOSUT’) (Saalbach *et al.*, 2014) versus wild-type Certo; (ii) ambient (380 ppm) versus elevated CO_2_ (550 ppm); (iii) 200 kg N ha^–1^ versus 300 kg N ha^–1^ fertilization; and (iv) no heat wave (0) versus application of a ‘typical’ heat wave according to De Boeck *et al*, (2010) (1). The design resulted in 16 treatment combinations, which were each tested with six replicates (96 samples in total); one repetition consisted of one pot with two plants each. The experiment allowed study of the effect of each factor on response variables related to plant phenology, yield-related traits, grain dimensions, and grain composition. Additionally, effects of interactions between factors on response variables were determined. The results were analysed by four-way ANOVA using OriginPro 8.1 software (www.originlab.de/) and the statistical software program R (www.r-project.org/).

### Plant growth

Seeds of winter wheat cv. Certo and HOSUT were germinated in greenhouses (20 °C) in trays with substrate mix compost, substrate 2 (www.klasmann-deilmann.com/), and sand (2:2:1) for 2 weeks, followed by 8 weeks of incubation in vernalization chambers (4 °C/4 °C, 8 h/16 h day/night regime). For the four-factorial experiment, plants were transferred to a CO_2_-equipped climate chamber system (Vötsch BioLine VB1514/S, Balingen, Germany) under controlled temperature, humidity, and photon flux density (PFD) involving two CO_2_ levels. Chamber conditions simulated the typical seasonal increments of day length, temperature, and light in South-Western Germany (Schmid *et al.*, 2016).

**Fig. 1.**
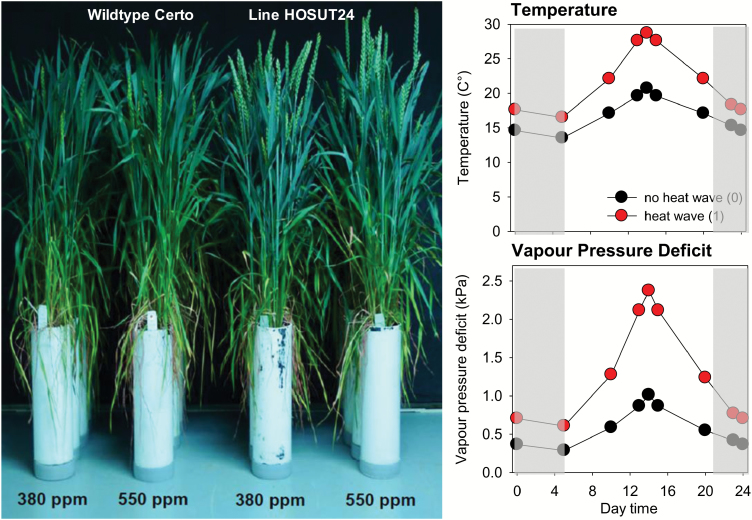
Wheat plants of Certo and HOSUT around the flowering stage as grown in the climate chambers; note developmental differences between Certo and HOSUT. (B) Heat wave temperature profile modulated with respect to duration, daytime temperature, and vapour pressure deficit according to typical conditions in Southern Germany. The heat wave started 2 days after flowering (DAF) until 11 DAF, covering early grain development. The grey shaded area depicts the dark period. (This figure is available in colour at *JXB* online.)

Two wheat plants were grown in one pot (10 cm diameter, 40 cm height; Fig. 1A) using substrate mixed from standard soil (Fruhstorfer Erde LD 80, HAWITA Group GmbH, Vechta, Germany) with washed river sand (Ø 0.3–2 mm) 1:2 (v:v), which resulted in an N supply of 200 kg ha^–1^. Exposure in the climate chambers was performed at ambient (380 ppm) and elevated (550 ppm) CO_2_, in combination with normal (200 kg N ha^–1^) and increased N (300 kg N ha^–1^) by adding nutrient solution using calcium nitrate tetrahydrate, under both standard climate and application of a heat wave. The heat wave was modulated with respect to duration and daytime air temperature and humidity according to typical conditions in Western Europe (Fig. 1B). The heat wave started 2 days after flowering (DAF) until 11 DAF, covering early grain development. Total water supply per pot was registered until final harvest, with watering performed at least every day. Potential water loss due to drainage was prevented by supplying each pot with a saucer and pouring drainage water back into the respective pot when necessary. Evaporation from the pots’ soil surface was not taken into account since we were interested in agronomic water use efficiency (WUE), which was calculated by relating water supply to aboveground biomass.

### Transgene expression analysis by quantitative real-time PCR (qRT-PCR)

Wheat plants were grown in greenhouses (Saalbach *et al.*, 2014), and total RNA was extracted using material of three biological replicates (Staroske *et al.*, 2016). First-strand cDNA was synthesized with an oligo(dT) primer and SuperScript III reverse transcriptase (Invitrogen). qRT-PCR was performed using Power Sybr^®^ Green PCR Mastermix, with 25 ng of amplified cDNA in each PCR. Using the HvSUT1-specific primers: forward, 5'-CGG GCG GTC GCA GCT CGC GTC TAT T-3'; and reverse, 5'-CAT ACA GTG ACT CTG ACC GGC ACA CA-3', qRT-PCR was performed with an ABI Prism 7900HT Sequence Detection System (Applied Biosystems), with 2 min at 50 °C, 40 cycles of 10 min at 95 °C, 15 s at 95 °C, and 1 min at 60 °C following a dissociation stage of 15 s at 95 °C, 15 s at 60 °C, and 15 s at 95 °C. Amplification efficiency was assessed with the LinRegPCR program. The wheat *actin* gene (accession no. AB181991) was used for normalization; *actin* primers were forward, 5'-GTG GAG GTT CTA CCA TGT TTC CTG-3'; and reverse, 5'-GCT AAG AGA GGC CAA AAT AGA GCC-3'.

### Cell number determination

Endosperms from the greenhouse plants, 4–18 d after fertilization, were prepared by hand dissection, and nuclei of five endosperms each of Certo and HOSUT were isolated by chopping with razor blades in 1 ml of nuclei isolation buffer (Galbraith *et al.*, 1983) supplemented with 1 μg ml^−1^ DAPI. Nuclei were counted on a ploidy analyser (PARTEC GmbH, http://www.partec.com/). As each cell harbours one nucleus, the number of counted nuclei directly reflects the cell number.

### Analysis of grain morphology, sucrose, starch, carbon, nitrogen, free amino acids, grain proteins, and microelements

TGW, grain width, length, and area of mature dry grains from the plants grown in the climate chambers were determined using the digital seed analyser MARVIN (www.gta-sensorik.com/). Mature grains were photographed using the Keyence VX 100K microscope unit (www.keyence.com/). A 5 g aliquot of grains per sample was ground using a ball mill to a fine powder. Sucrose and starch were determined by coupled enzyme assays in duplicate as described (Rolletschek *et al.*, 2002). Relative contents of total C and N were measured using an elemental analyser (Vario Microcube; Elementaranalysensysteme, http://www.elementar.de/).

Macro- and microelements in mature grains were measured by inductively coupled plasma optical emission spectrometry (ICP-OES, iCAP 6000, Thermo Fisher Scientific, Germany) combined with the CETAC ASXPRESS™ *PLUS* rapid sample introduction system, and a CETAC autosampler (CETAC Technologies, Omaha, NE, USA). A 50 mg aliquot of dried and ground samples was digested with 2 ml of 69% nitric acid (HNO_3_) (Bernd Kraft GmbH, Germany) using the high performance microwave reactor UltraClave IV from MLS GmbH (Germany). Digested samples were filled up to 15 ml final volume with deionized water from the Milli-Q^®^ Reference System, Merck (Germany). Element standards (Bernd Kraft multi-element standard solution, Germany) Mo, Na, Mg, P, S, Ca, Mn, Fe, Cu, Zn, and K were used as external standards, and Yttrium (ICP Standard Certipur^®^ Merck Germany) was used as an internal standard for matrix correction.

Free amino acid concentrations were measured by ultra performance liquid chromatography (UPLC) using a Waters Acquity UPLC^®^ system (www.waters.com/). Extraction and analysis were performed as described (Thiel *et al.*, 2009; Kohl *et al.*, 2015). For the analysis of the protein distribution, 50 g of mature grains of each sample of the 16 treatment combinations were ground and analysed each with three technical replicates as described (Saalbach *et al.*, 2014), with the following modification of the HPLC system: instrument, Jasco XLC (Jasco, Germany); column: Acclaim™ 300 C_18_ (3 µm, 30 nm, 2.1 × 150 mm, Thermo Fisher Scientific, Germany); temperature, 60 °C; injection volume, 5 µl of albumin/globulin, 10 µl of gliadin, and 20 µl of glutenin extracts; elution solvents, (A) water/trifluoroacetic acid (TFA) (999 + 1, v/v), (B) acetonitrile/TFA (999 + 1, v/v); gradient for albumins/globulins, 0 min 0% B, 0.5 min 20% B, 7 min 60% B, 7.1–11 min 90% B, 11.1–17 min 0% B; gradient for gliadins and glutenins, 0 min 0% B, 0.5 min 24% B, 20 min 56% B, 20.1–24.1 min 90% B, 24.2–30 min 0% B; flow rate, 0.2 ml min^–1^; detection, UV absorbance at 210 nm. PWG-gliadin (Van Eckert *et al.*, 2006) dissolved in 60% (v/v) ethanol was used for external calibration in the range of 11.6–46.6 µg to calculate the protein contents of the fractions.

### Crop performance

The date of flowering of the main stem was registered daily using the BBCH decimal code (Tottman, 1987) and is presented as days after planting. The final harvest was performed when at least 50% of all pots per treatment reached full maturity (DC89). Canopy height as well as tiller and ear number per pot were measured at maturity. Aboveground biomass of wheat plants was harvested and separated into leaves, stems, and ears. Vegetative and generative biomass fractions were oven-dried at 60 °C and 30 °C, respectively. The dry weight of all biomass fractions was determined until they reached constant weight. After careful threshing to retain all grains, the harvest index, grain number per pot, and grain number per ear were estimated. Total water supply per pot was registered until final harvest, and related to aboveground biomass in order to calculate the WUE.

## Results

### HOSUT grains display increased endosperm cell number

**Fig. 2.**
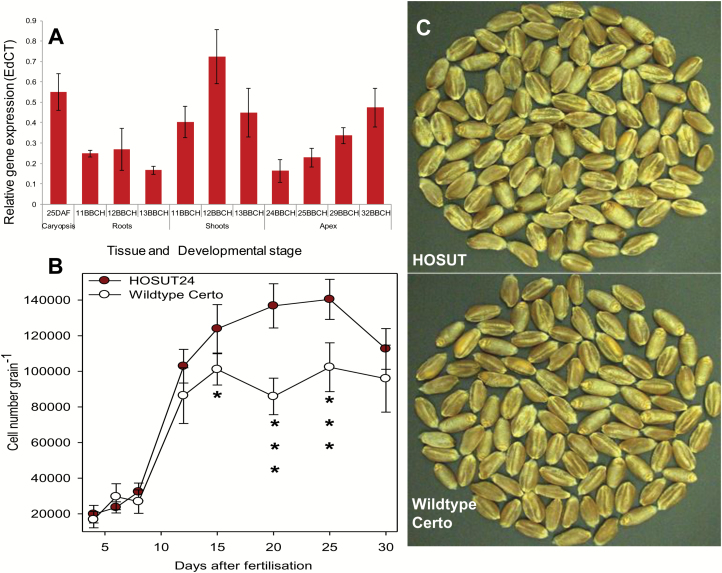
(A) *HvSUT1* transgene expression in different tissues of the HOSUT line on the basis of quantitative real-time PCR. Development was recorded using the BBCH scale, BBCH 11, 12, 13, one-, two-, and three-leaf stage, respectively; BBCH 24, 25, tillering stage; 29, end of tillering; 32, node 2 at least 2 cm above node 1. (B) Endosperm cell number per grain for HOSUT24 and Certo. Data are means ±SD, *n*=6, significant differences according to *t*-test, *P<0.05, ***P<0.001. (C) Mature grains of HOSUT and Certo. (This figure is available in colour at *JXB* online.)

Wheat transformation and generation of lines was described in Saalbach *et al.* (2014). The qRT-PCR-based gene expression analysis revealed that the *HvSUT1* transgene was strongly expressed in caryopses at 25 d after fertilization but also in young shoots, roots, and apices (Fig. 2A). Previous results showed that HOSUT grains of independent lines showed increased size compared with Certo (Saalbach *et al.*, 2014). Fluorescence-based flow cytometry was used to analyse cell numbers during endosperm development. The results revealed higher cell numbers in the HOSUT endosperm from 15 d to 25 d after fertilization (Fig. 2B), which indicated a possible assimilate effect on endosperm cell proliferation and might have increased mature grain size of the HOSUT plants (Fig. 2C). Reduced cell number in HOSUT endosperm at 30 d after fertilization could be due to earlier maturation coupled with endosperm cell death (Domínguez and Cejudo, 2014).

### Crop performance

The flowering start date for Certo was 92.5 d after planting under ambient CO_2_ conditions and 91 d after planting under elevated CO_2_. Thus elevated CO_2_ led to earlier flowering of Certo by 1.5 d (*P*<0.0001). The flowering start date for HOSUT, under both ambient and elevated CO_2_, was 90 d after planting, indicating that in contrast to Certo the high CO_2_ treatment did not shift the start of flowering of HOSUT. Furthermore, HOSUT under ambient and elevated CO_2_ started flowering 2.5 d and 1 d earlier than Certo, respectively (*P*<0.0001).

**Fig. 3.**
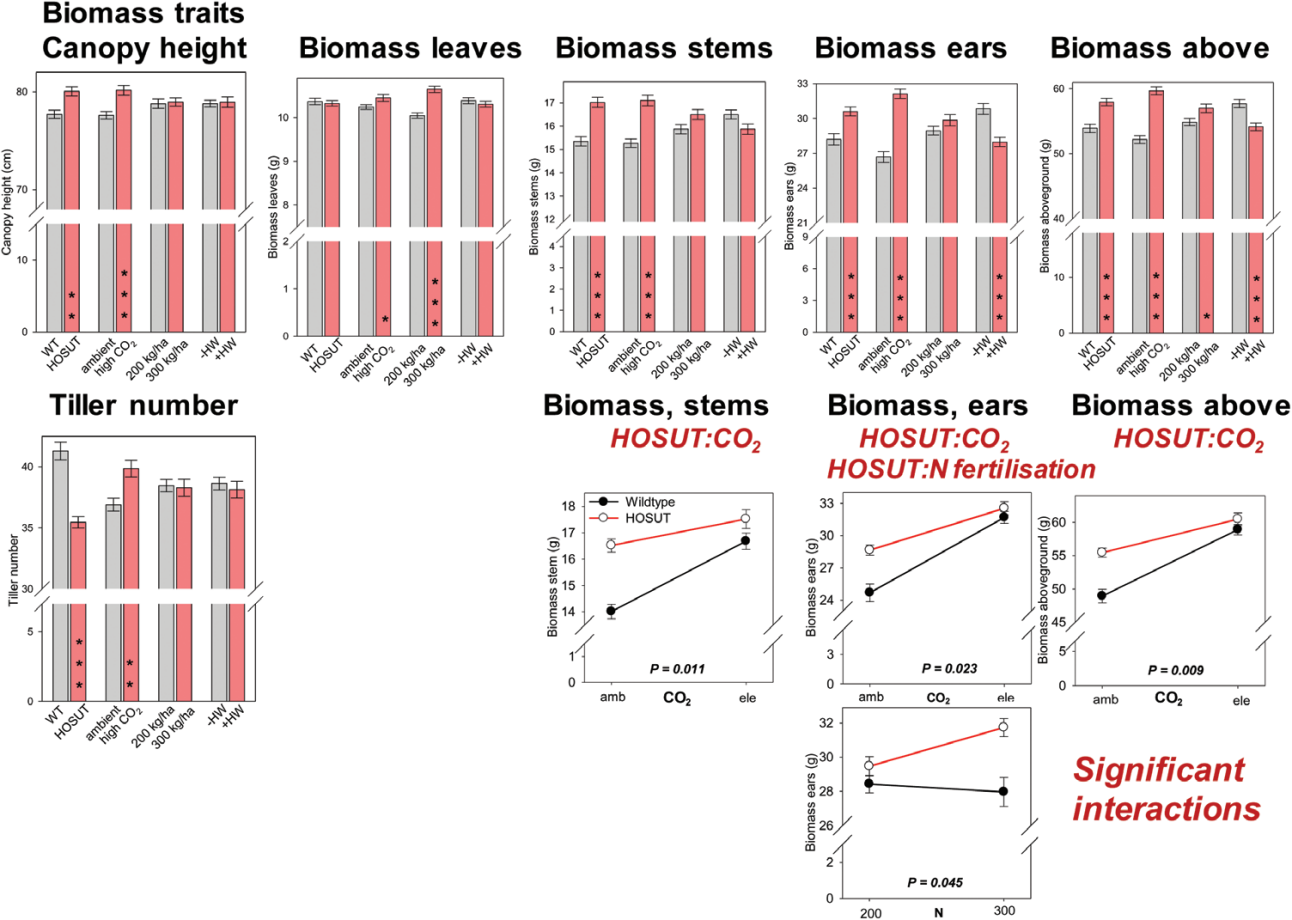
Bar charts: the factors line, CO_2_, N fertilization, and heat wave influencing the biomass traits canopy height, biomass of leaves, stems, ears, and aboveground biomass. Bars represent the mean across all other treatments and are means ±SE, *n*=48, significant differences according to four-way ANOVA, *P<0.05; **P<0.01, ***P<0.001. Line plots: significant interactions between the factors line:CO_2_ and line:N fertilization; data points are means ±SE, *n*=24. (This figure is available in colour at *JXB* online.)

HOSUT effects and treatment with elevated CO_2_ similarly affected plant performance by increasing canopy height and organ biomass, especially that of stems and ears. The number of tillers was clearly lower for HOSUT compared with Certo, but was higher under elevated CO_2_ compared with ambient conditions. High N fertilization led to higher aboveground biomass, particularly for leaves. Heat wave treatment during early grain filling decreased aboveground biomass, especially that of ears (Fig. 3). All *P*-values according to the four-way ANOVA analysis are given in Table 1 and in Supplementary Table S1 at *JXB* online.

**Table 1.**
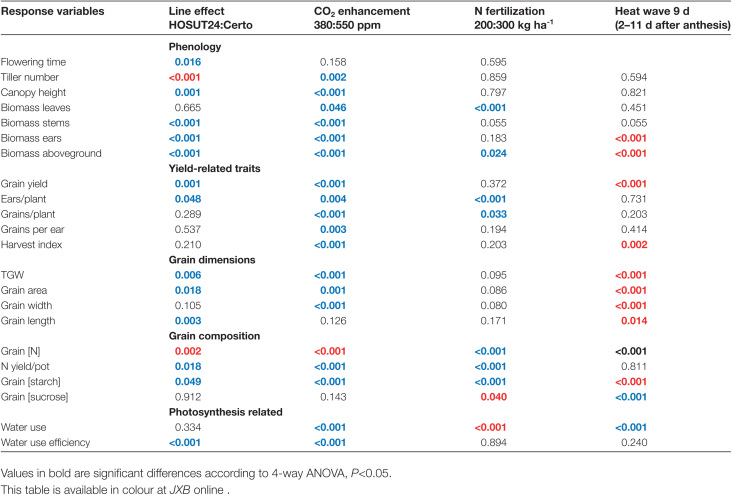
*P*-values signifying the influence of factors HOSUT, CO_2_, N fertilization, and heat wave (upper line) on response variables

HOSUT and Certo interacted significantly differently with CO_2_ treatment for the traits stem, ear, and aboveground biomass. Under high CO_2_ conditions, there was a stronger stimulatory response for Certo compared with HOSUT. A line effect was also visible for N fertilization, revealing that for the trait ear biomass, high N fertilization was stimulatory for HOSUT only and not for Certo (Fig. 3).

### Yield-related traits

**Fig. 4.**
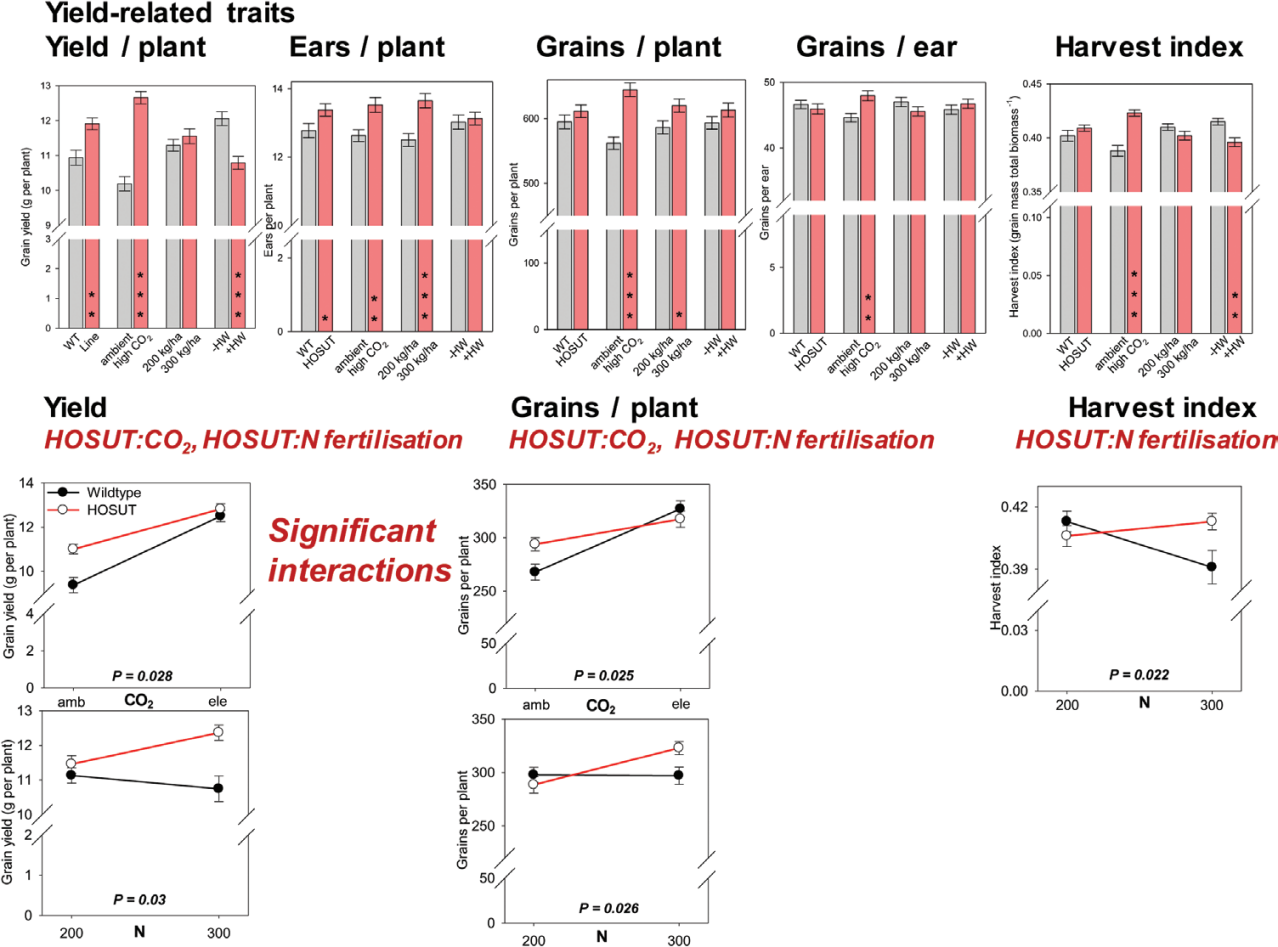
Bar charts: the factors line, CO_2_, N fertilization, and heat wave influencing the yield-related traits grain yield, ears per pot, grain number per pot, grain number per ear, and harvest index in line HOSUT and wild-type Certo. Bars represent the mean across all other treatments and are means ±SE, *n*=48, significant differences according to four-way ANOVA, *P<0.05; **P<0.01, ***P<0.001. Line plots: significant interactions between the factors line:CO_2_ and line:N fertilization, data points are means ±SE, *n*=24. (This figure is available in colour at *JXB* online.)

Grain yield and ear number per pot were higher for HOSUT compared with Certo. Elevated CO_2_ concentration increased the yield-related traits grain weight, ear and grain numbers per pot, grain number per ear, and harvest index. High N fertilization raised ear and grain numbers per pot. Application of a heat wave decreased grain yield and harvest index (Fig. 4).

For the traits grain yield and grain number per pot, line×CO_2_ treatment interactions were detected: at increasing CO_2_, these traits increased more strongly in Certo than in HOSUT. There were also a line×N fertilization interaction for these traits since there was a clear increase in grain yield and grain number per pot at high N supply for HOSUT but not for Certo. For harvest index, a line×N fertilization interaction occurred with a slight increase for HOSUT at high N supply but a strong decrease for Certo (Fig. 4).

**Fig. 5.**
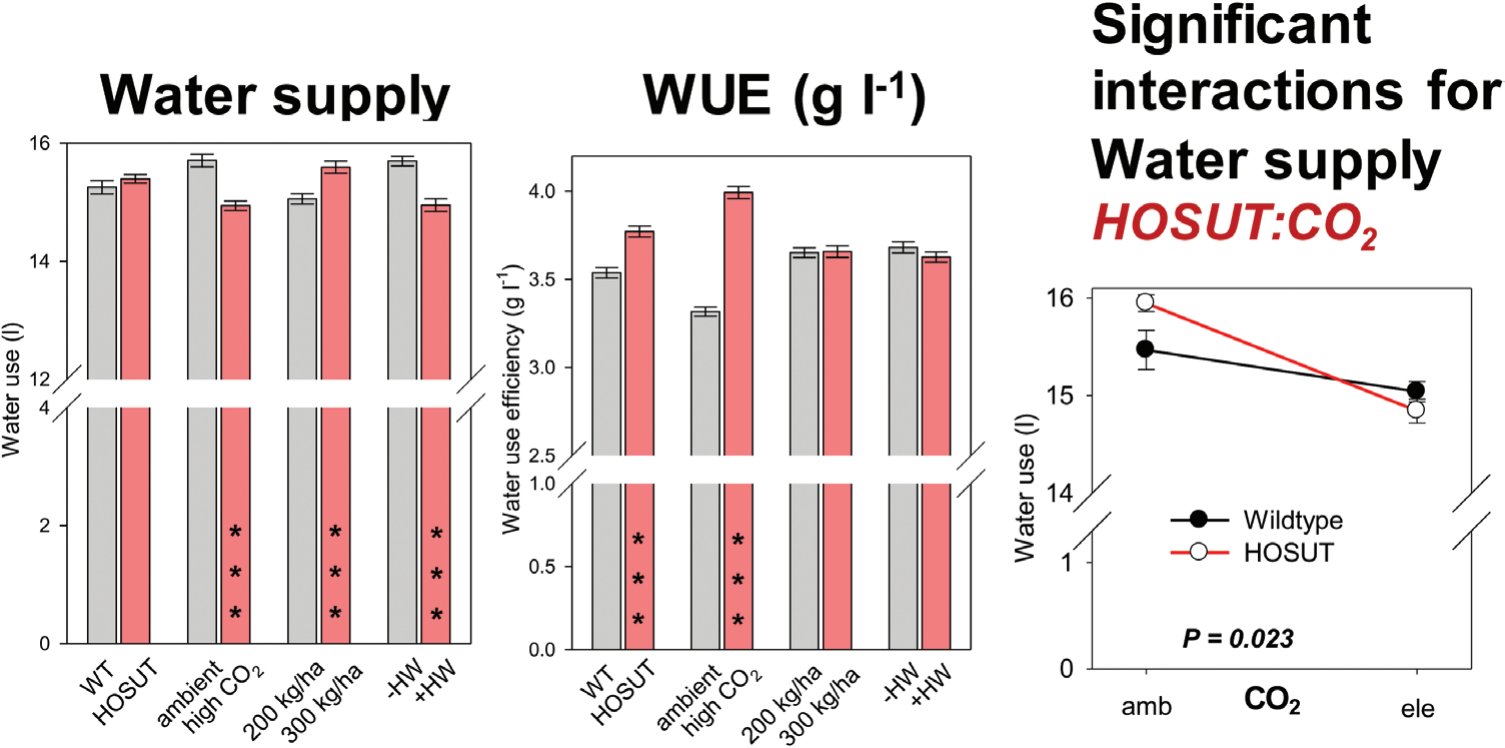
Bar charts: the factors line, CO_2_, N fertilization, and heat wave influencing water supply and water use efficiency (WUE). Bars represent the mean across all other treatments and are means ±SE *n*=48, significant differences according to four-way ANOVA, ***P<0.001. Line plot: significant interaction between the factors line:CO_2_ and line:N fertilization. Data points are means ±SE, *n*=24. (This figure is available in colour at JXB online.)

Water supply (i.e. the cumulative amount of water provided to the pots to keep plants well watered) did not differ between lines, was lower at elevated CO_2_, higher at high N supply, and lower for plants grown with the heat wave. With respect to WUE, HOSUT performed better than Certo. Elevated CO_2_ strongly increased WUE whereas WUE remained unchanged due to high N supply or the heat wave application (Fig. 5). A line×CO_2_ treatment interaction was present for water supply since the water-saving effect at elevated CO_2_ was much more pronounced for HOSUT than for Certo (Fig. 5).

### Grain dimensions and composition

**Fig. 6.**
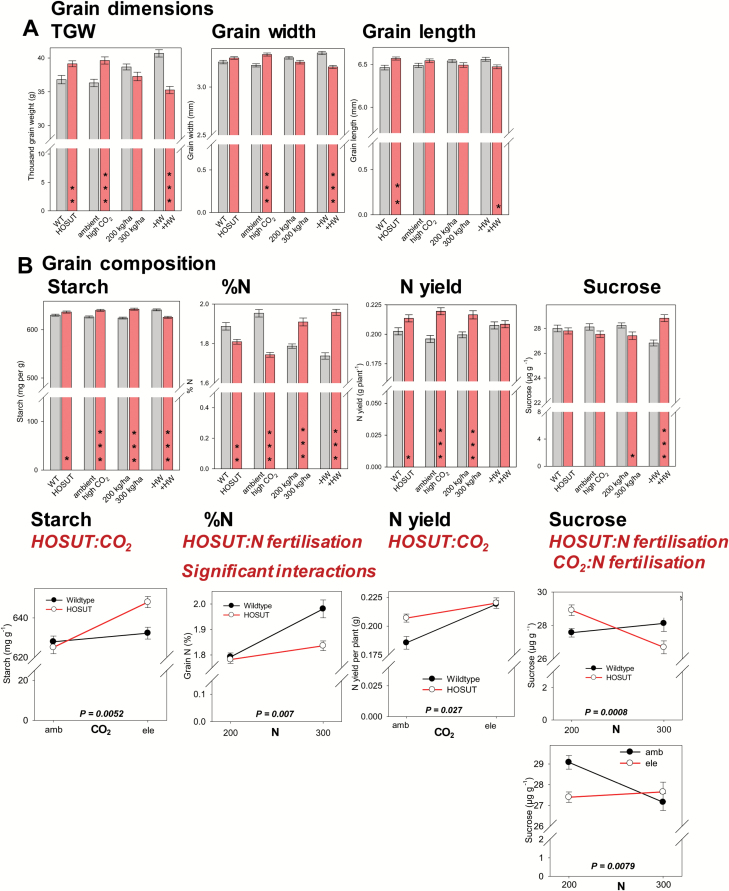
(A) Bar charts: thefactors line, CO_2_, N fertilization, and heat wave influencing grain dimensions, thousand grain weight (TGW), grain width. and grain length; bars are means ±SE, *n*=48, significant differences according to four-way ANOVA, *P<0.05; **P<0.01, ***P<0.001. (B) Bar charts: the factors line, CO_2_, N fertilization, and heat wave influencing grain composition, starch, percentage N, N yield, and sucrose. Bars represent the mean across all other treatments and are means ±SE *n*=48, significant differences according to four-way ANOVA, *P<0.05; **P<0.01, ***P<0.001. Line plots: significant interactions between the factors line:CO_2_, line:N fertilization, and CO_2_:N fertilization. Data points are means ±SE, *n*=24. (This figure is available in colour at *JXB* online.)

TGW and grain length were larger in HOSUT than in Certo. Growth under elevated CO_2_ increased TGW and grain width, whereas the heat wave application strongly decreased TGW, grain width, and length (Fig. 6A).

Starch concentration was higher and grain N percentages were lower in grains of HOSUT compared with Certo. However, total N yield was superior for HOSUT, probably due to its higher TGW and ear number, which possibly more than compensated the lower grain N concentration. Elevated CO_2_ treatment increased grain starch, but decreased grain N concentration. High N fertilization increased grain starch and N concentration as well as total N yield of grains. The heat wave treatment decreased grain starch, but increased grain N concentration. Grain sucrose concentration was lower at high N fertilization. The heat wave application strongly increased grain sucrose concentration, possibly due to impaired sucrose to starch conversion (Fig. 6B).

Several treatment interactions were detected for grain composition traits. Grain starch showed a strong increase at elevated CO_2_ in HOSUT, but no significant response in Certo. The grain N concentration showed a strong positive response to additional N supply in Certo but much less so in HOSUT. For total N yield, an interactive response existed between line and CO_2_ enrichment. At ambient CO_2_, N yield was much higher in HOSUT than in Certo, but this advantage was lost at elevated CO_2_. N supply and line had interactive effects on grain sucrose concentration which decreased in HOSUT grains but not in Certo grains when additional N was supplied. Grain sucrose levels were also affected differently by CO_2_ enrichment and N fertilization. At elevated CO_2_, sucrose remained low at both N fertilization levels, but was significantly higher at ambient CO_2_ and normal N fertilization (Fig. 6B).

The analysis of microelements in mature grains revealed that HOSUT did not differ greatly from the wild type with respect to grain nutrient element concentrations. Significant line differences were found only for Zn, with higher, and for K, with lower values in HOSUT grains. In contrast, CO_2_ enrichment strongly decreased all elements, except Mo. High N fertilization increased S, Mo, and Ca. The heat wave treatment during early grain development increased the concentrations of all elements, except Mo, which was probably due to combined effects of reduced grain weight and lower starch concentration (Table 2; Supplementary Table S2A).

**Table 2.**
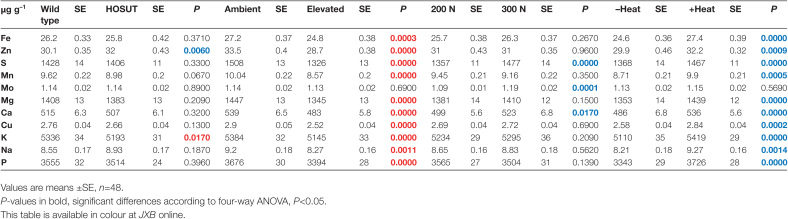
Factors HOSUT, CO_2_, N fertilization, and heat wave influencing the concentration of nutrient elements in mature grains of Certo and HOSUT

Free amino acid concentrations in mature grains were lower in HOSUT compared with Certo and were diminished due to elevated CO_2._ The amino acids concerned were asparagine, aspartate, glutamine, and glycine. High N fertilization and growth under the heat wave increased several amino acids in the grains (Table 3; Supplementary Table S2B). However, again combined effects of reduced grain weight and lower starch concentration could be causal for the response to the heat wave.

**Table 3.**
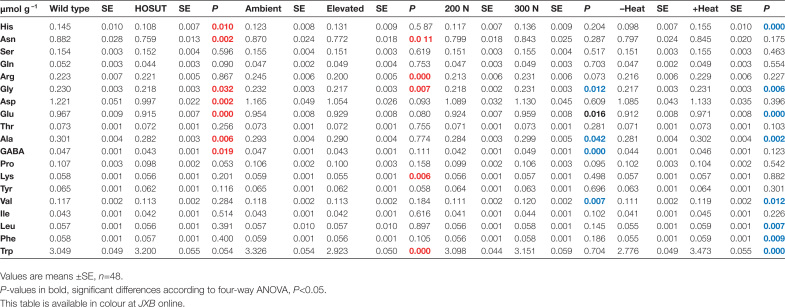
Factors HOSUT, CO_2_, N fertilization, and heat wave influencing the concentration of free amino acids in mature grains of Certo and HOSUT

The analysis of the storage proteins in mature grains showed that HOSUT manifested decreased crude protein, extractable proteins, gluten, and gliadins by 5–8% compared with Certo. Albumins/globulins, total glutenins, and low molecular weight glutenin subunits γ- and ωb-gliadins were not significantly different between the lines. Elevated CO_2_ decreased all protein classes by 10–14%, except albumins/globulins and ωb-gliadins. High N fertilization increased the concentration of all protein fractions and types by >10%, except albumins/globulins. When grown under the heat wave, mature grains exhibited increased concentrations of all proteins by 11–20%, except ωb-gliadins (Table 4; Supplementary Table S2C).

**Table 4.**
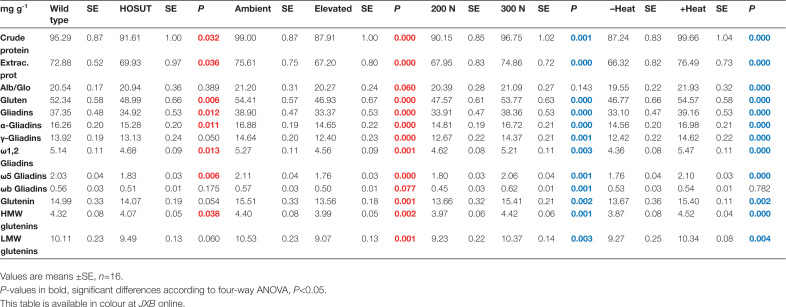
Factors HOSUT, CO_2_, N fertilization, and heat wave influencing the concentration of crude protein, extractable protein, albumins/globulins, gluten, γ-gliadins, ω-1,2 gliadins, ω5-gliadins, ωb-gliadins, glutenins, high molecular weight glutenin subunits (HMW-GS), and low molecular weight glutenin subunits (LMW-GS) in mature grains of Certo and HOSUT

## Discussion

Optimizing assimilate partitioning to the spike and increasing harvest index are of high priority for improving the yield potential of wheat (Reynolds *et al.*, 2009). Unloading of assimilates into grains represents a potential bottleneck, and increasing transport activities might therefore improve grain sink strength (Ruan, 2014). We showed that ectopic expression of the barley *HvSUT1* sucrose transporter gene in winter wheat increased sink activity under semi-controlled conditions and led to significantly increased grain yield compared with Certo, mainly due to higher TGW and increased ear number per plant (Saalbach *et al.*, 2014). Thus, HOSUT plants are models with improved assimilate supply to grains, leading to higher grain yield.

While in HOSUT plants sink activity has been increased, elevated CO_2_ signifies increased source activity, which in many plants stimulates photosynthesis and regularly induces faster growth and biomass accumulation (Amthor, 2001; Jablonski *et al.*, 2002). This study analysed how increased sink activity of HOSUT responds to source stimulation and whether additional C provided by CO_2_ enrichment can be better used by HOSUT. In parallel, the effects of a heat wave during early grain development and influences from high N fertilization were analysed.

### The HOSUT line has favourable traits at the whole-plant level

The *HvSUT1* transgene is expressed in developing caryopses but, somewhat unexpectedly, also in other tissues, preferentially in young vegetative sink organs (Fig. 2A). It was shown earlier that the *Hordein B1* promoter controls a seed storage protein gene in the endosperm (Marris *et al.*, 1988). However, promoters across different cereals may change their tissue specificity (Furtado *et al.*, 2009), indicating that the barley *Hordein B1* promoter in wheat is not solely grain specific, and altered sucrose transport potential and partitioning can be anticipated in other organs as well. This may explain the changed performance of HOSUT with respect to lower tiller numbers, higher number of ears per pot, and higher aboveground biomass. If the transgene is already active in young organs, this might lead to more ears. Later on, the high grain sink strength could lead to an earlier senescence of existing tillers, which would reduce the number of existing tillers at maturity.

While flowering time was accelerated only in Certo by elevated CO_2_, HOSUT plants flowered earlier under both ambient and elevated CO_2_ concentration compared with Certo. Sucrose is important to stimulate flowering induction and, accordingly, changed expression of sucrose transporters alters flowering time, indicating that sucrose translocation and distribution are critical (Hackel *et al.*, 2006; Sivitz *et al.*, 2007; Chincinska *et al.*, 2008). Early flowering is also reported under CO_2_ enrichment (Reekie *et al.*, 1994; Springer *et al.*, 2008; May *et al.*, 2013), which likewise may be an effect of improved assimilate supply. The *HvSUT1* transgene is expressed in apices (Fig. 2A). Thus, sucrose effects within the apex can be expected such as stimulation of cell proliferation resulting in general acceleration of development. Subsequently, this promotes flowering time in HOSUT plants, but without further acceleration by CO_2_ enrichment.

### HOSUT effects and CO_2_ enrichment are partially overlapping

Increased TGW is a stable characteristic of HOSUT and has been repeatedly observed across different years and lines (Weichert *et al.*, 2010; Saalbach *et al.*, 2014). From early grain filling onwards, HOSUT endosperm cell number exceeds that of Certo (Fig. 2B). Sugar signalling and assimilate supply affect cell proliferation together with many other factors, which may integrate and communicate sucrose flux, or the potential of sucrose flux (Dante *et al.*, 2014). Carbon source availability as sucrose determines cell division by controlling the expression of D-type cyclins in Arabidopsis (Riou-Khamlichi *et al.*, 2000). Accordingly, endosperm cell proliferation in wheat is regulated by assimilate supply (Brocklehurst, 1977; Singh and Jenner, 1984; Ruan, 2014). It is concluded that increased HOSUT endosperm cell number is responsible for higher grain size.

Both factors, intrinsic HOSUT traits and CO_2_ enrichment, positively affected phenological and yield-related traits in a similar manner, as was shown for canopy height, biomass of stems and ears, total aboveground biomass, grain yield, ears per plant, TGW, grain starch concentration, and WUE. However, the two factors differ in their mode of action. While CO_2_ enrichment frequently stimulates biomass accumulation by improving C assimilation and thereby increasing source strength, the HOSUT transgene potentially fuels sucrose transport and partitioning mainly in grains and young sink tissues, and thus increases sink strength.

While for some phenotypic changes, HOSUT effects mimicked those of CO_2_ enrichment, clear differences accounted for others. Tiller number was increased due to CO_2_ enrichment, but was decreased in HOSUT compared with Certo. Nevertheless, ear number per plant was increased by both treatments. This confirms that elevated CO_2_ and HOSUT effects at least partially function in a different context.

Grain nutrient element concentrations were in general lower at elevated CO_2_, but were not lower (Table 2) or were even increased in HOSUT grains compared with Certo (Saalbach *et al.*, 2014). Thus, the effects of gene expression in the HOSUT line improved grain nutrient element concentration relative to CO_2_ enrichment. Possibly, amended transport and/or allocation could play a role because it has been suggested that feed-forward control of microelement loading is regulated by sucrose flux into the endosperm and therefore may respond to increased grain sink strength of the HOSUT line (Stomph *et al.*, 2011). The transfer route of nutrient elements into grains resembles that of sucrose (Pearson *et al.*, 1998). Higher Zn contents, as seen in the HOSUT grains (Table 2), might be due to improved sucrose uptake capacities, supporting the suggestion that grain sink strength is important for micronutrient delivery (Stomph *et al.*, 2011). Another factor could be a possibly increased mass flow due to HOSUT effects. A relationship between transpiration and nutrient acquisition has been suggested (McGrath and Lobell, 2013), showing that higher mass flow occurs upon increased water use/transpiration, which could improve nutrient acquisition. Since mineral element depletion in grains due to increasing atmospheric CO_2_ concentrations might represent a threat to human nutrition in the future (Högy and Fangmeier, 2008; Myers *et al.*, 2014), this trait of HOSUT should be considered when dealing with hidden hunger problems.

### HOSUT is saturated in response to CO_2_ fertilization

HOSUT and Certo responded significantly differently to CO_2_ treatment with respect to biomass of stems and ears, total aboveground biomass, grain yield, N yield, and grains per plant. For both HOSUT and Certo, all these traits reacted positively to CO_2_ enrichment. However, the response was significantly more pronounced for Certo. CO_2_ enrichment was less effective in HOSUT, indicating that it is already optimized according to assimilate translocation to grains. Thus, while Certo could be strongly promoted by elevated CO_2_, generating additional sinks such as stems and ears, HOSUT was more saturated and responded only weakly. The fact that HOSUT did not strongly react directs attention to other factors, which may be limiting for grain filling in wheat. While sucrose transporters could affect assimilate loading and unloading, other limiting factors can be critical such as development of the vascular system and/or short-distance transport within the spike, rachis, and spikelet.

Higher individual grain sink strength in HOSUT is accompanied by lower grain number per spike, a trait which strongly depends on assimilates allocated to the spike (Ghiglione *et al.*, 2008). A negative relationship between grain size and grain number per spike has been observed earlier for HOSUT (Saalbach *et al.*, 2014) and could indicate limited assimilate supply/transport at the spike level. Accordingly, we conclude that higher individual grain sink strength cannot be fully balanced by assimilate transport into HOSUT ears.

The line responses to CO_2_ enrichment revealed that increased starch levels at elevated CO_2_ occurred only for HOSUT but not for Certo (Fig. 6B). Thus, grain starch of HOSUT responded more strongly to CO_2_ than Certo, indicating again that translocation processes/grain sink strength are more optimized in HOSUT compared with Certo. Subsequently, the additional C provided from CO_2_ enrichment could be more efficiently used, especially in the grains.

### HOSUT responds more strongly to high N fertilization compared with Certo

Both HOSUT effects and CO_2_ enrichment decreased the concentrations of free amino acids, storage proteins, and grain N in a similar way, although the effect was stronger for CO_2_ enrichment (Fig. 6B; Table 4). Thus, yield increases, due to either HOSUT effects or CO_2_ enrichment, were strongly associated with loss of grain quality, expressed as a percentage of grain N (Supplementary Fig. S1). It has been shown that CO_2_ enrichment frequently reduces N concentrations in plants (Taub *et al.*, 2008). The mechanisms responsible are dilution effects by increased production of non-structural carbohydrates, decreased root N uptake, reduced transpiration and N uptake with water mass flow from the soil, as well as improved photosynthetic N use efficiency by less N investment in Rubisco. Our results show that CO_2_ and HOSUT effects exert analogous C:N imbalances. However, line responses to N fertilization for ear biomass, grain yield, grains per pot, and harvest index indicate N stimulation preferentially for HOSUT. This could be due to the fact that HOSUT is optimized for assimilate transfer/mass flow especially towards grains and may profit from the additional N with respect to ears and grains (Taub and Wang, 2008; Chapman *et al.*, 2012). This could finally improve grain yield, ear biomass, and grain number per plant. On the other hand, vegetative growth was preferentially stimulated in Certo by high N fertilization relative to HOSUT. The optimized N use is also underpinned by the fact that high N fertilization decreased the harvest index in Certo relative to HOSUT. High N fertilization in Certo stimulated vegetative growth and decreased the harvest index, whereas in HOSUT N could be used more efficiently due to improved transport to sinks, not only for sucrose but also for N.

The heat wave at early grain filling decreased aboveground and ear biomass, grain yield, harvest index, grain size parameters, water use, and grain starch concentration, and at the same time increased grain sucrose concentration, but did not alter N yield for both HOSUT and Certo. Even a moderate heat wave during early grain development, which is quite common in spring or early summer in Western Europe, strongly affected grain developmental parameters, which finally decreased yield by ~15%. The heat wave strongly affected starch biosynthesis and/or conversion of sucrose to starch, which is reflected by an increased sucrose to starch ratio in the grains (Barnabás *et al.*, 2008; Mangelsen *et al.*, 2011; Phan *et al.*, 2013).

The increase of the concentrations of all storage proteins except albumins/globulins (Table 4) after additional N fertilization clearly showed that this treatment had the expected effect. An increased content of gluten proteins is associated with improved technological properties (quality) of the flour. The HOSUT line and the CO_2_ effects on the protein distribution confirmed earlier studies that looked at these effects separately (Högy *et al.*, 2009; Saalbach *et al.*, 2014).

## Conclusions

The HOSUT line expressing a sucrose transporter is potentially enhanced in sucrose transport and shows a superior performance for many yield-related traits compared with Certo (Table 1). Our results reveal that both HOSUT and CO_2_ enrichment similarly improve a wide range of phenotypic and yield-related traits although associated with lower grain quality. Responses to elevated CO_2_ are more pronounced for Certo and less effective for HOSUT. This suggests that HOSUT is already optimized and may benefit only weakly from any future CO_2_ increase. The fact that HOSUT cannot use additional C from source stimulation as efficiently as Certo points to other rate-limiting factors for grain filling, such as short-distance transport at the spike level. Obviously the improved sink strength of HOSUT diminishes a positive CO_2_ effect on grain yield. While both factors, HOSUT and elevated CO_2_, diminish grain protein concentration, HOSUT benefits more strongly from high N fertilization with respect to grain yield, grain number per plant, and ear biomass. This might point to a basic improvement in assimilate transfer, especially to grains. Relatively high N fertilization is necessary to develop the HOSUT yield advantage. Application of a moderate heat wave at early grain filling considerably diminishes grain size, yield, and starch concentration without apparent interaction with line or CO_2_ effects.

## Supplementary data

Supplementary data are available at *JXB* online.

Table S1. *P*-values signifying the influence of factors HOSUT, CO_2_, N fertilization, and heat wave on response variables and all interactions.

Table S2. A, B, and C, data from Tables 2, 3, and 4: concentration of nutrient elements, free amino acids, and storage protein classes, given as percentages.

Fig. S1. Correlation between grain yield and grain quality (%N).

## Conflict of interests

There is no conflict of interest concerning this manuscript.

## Supplementary Material

Supplementary_Tables_Figure_S1
