# Supplementary material for: Grain yield and quality responses of wheat expressing a barley sucrose transporter to combined climate change factors
Source: J Exp Bot. 2017 Oct 21;68(20):5511–25. doi: 10.1093/jxb/erx366 (PMC5853912; doi:10.1093/jxb/erx366)
Supplement: Supplementary_Tables_Figure_S1 [file erx366_suppl_supplementary_tables_figure_s1.pdf]

Table S1, P-values signifying the influence of factors HOSUT, CO_2, N fertilisation and heat wave on response variables (left row), and all interactions.

P - values	HOSUT	CO_2	N	HW	HOSUT:CO_2	HOSUT:N	HOSUT:HW	CO_2:N	CO_2:HW	N:HW	HOSUT:CO_2:N	HOSUT:CO_2:HW	HOSUT:N:HW	CO_2:N:HW	HOSUT:CO_2:N:HW
Flowering time	0.016	0.158	0.595												
Tiller number	<0.001	0.002	0.859	0.594	0.756	0.003	0.722	0.756	<0.001	0.029	0.451	0.451	0.594	0.756	0.005
Canopy height	0.001	<0.001	0.797	0.821	0.964	0.916	0.916	0.441	0.964	0.234	0.108	0.940	0.916	0.246	0.258
Biomass leaves	0.665	0.046	<0.001	0.451	0.427	0.072	0.086	0.233	0.340	0.167	0.043	0.161	0.458	0.256	0.411
Biomass stems	<0.001	<0.001	0.055	0.055	0.011	0.404	0.324	0.521	0.555	0.432	0.919	0.513	0.784	0.941	0.692
Biomass ears	<0.001	<0.001	0.183	<0.001	0.023	0.046	0.404	0.316	0.959	0.328	0.464	0.351	0.598	0.912	0.818
Biomass aboveground	<0.001	<0.001	0.024	<0.001	0.009	0.124	0.257	0.281	0.785	0.409	0.736	0.291	0.694	0.942	0.832
Grain yield	0.001	<0.001	0.372	<0.001	0.028	0.030	0.157	0.342	0.843	0.617	0.283	0.258	0.639	0.726	0.942
Ears/plant	0.048	0.004	<0.001	0.731	0.630	0.048	0.372	0.449	0.945	0.945	0.630	0.244	0.945	0.836	0.731
Grains/plant	0.289	<0.001	0.033	0.203	0.025	0.026	0.566	0.226	0.468	0.538	0.909	0.039	0.527	0.665	0.582
Grains/ear	0.537	0.003	0.194	0.414	0.042	0.440	0.825	0.617	0.381	0.628	0.453	0.253	0.559	0.777	0.356
Harvest index	0.210	<0.001	0.203	0.002	0.147	0.022	0.378	0.524	0.279	0.952	0.258	0.373	0.560	0.655	0.672
Thoisand grain weight	0.006	<0.001	0.095	<0.001	0.626	0.997	0.456	0.832	0.845	0.889	0.377	0.325	0.991	0.873	0.351
Grain area	0.018	0.001	0.086	<0.001	0.694	0.839	0.538	0.804	0.654	0.934	0.301	0.556	0.848	0.778	0.428
Grain width	0.105	<0.001	0.080	<0.001	0.599	0.852	0.468	0.684	0.739	0.706	0.384	0.441	0.808	0.815	0.303
Grain length	0.003	0.126	0.171	0.014	1.000	0.695	0.567	0.917	0.540	0.767	0.246	0.961	0.934	0.574	0.812
Grain [N]	0.002	<0.001	<0.001	<0.001	0.094	0.007	0.755	0.205	0.086	0.382	0.380	0.984	0.407	0.778	0.248
N yield/plant	0.018	<0.001	<0.001	0.811	0.027	0.250	0.336	0.415	0.972	0.962	0.932	0.139	0.741	0.937	0.598
Grain [starch]	0.049	<0.001	<0.001	<0.001	0.005	0.118	0.302	0.328	0.098	0.269	0.546	0.414	0.255	0.313	0.693
Grain [sucrose]	0.912	0.143	0.040	<0.001	0.654	<0.001	0.639	0.008	0.311	0.900	0.007	0.659	0.028	0.983	0.618
Water use	0.334	<0.001	<0.001	<0.001	0.023	0.727	0.730	0.188	0.837	0.162	0.774	0.469	0.951	0.214	0.870
Water use efficiency	<0.001	<0.001	0.894	0.240	0.072	0.082	0.289	0.832	0.886	0.887	0.783	0.366	0.643	0.451	0.819

Table S2, (A), data from Table 2, concentration of nutrient-elements, expressed as percentages

ratio [%]	HOSUT / wildtype			elevated / ambient			300N / 200N			+heat / -heat		
	Mean	SE	*P*	Mean	SE	*P*	Mean	SE	*P*	Mean	SE	*P*
**Fe**	98.5	2.0	0.3710	91.2	1.9	0.0003	102.3	2.1	0.2670	111.4	2.3	0.0000
**Zn**	106.3	1.9	0.0060	85.7	1.5	0.0000	100.0	1.8	0.9600	107.7	2.0	0.0009
**S**	98.5	1.2	0.3300	87.9	1.1	0.0000	108.8	1.4	0.0000	107.2	1.4	0.0000
**Mn**	93.3	3.0	0.0670	85.4	2.7	0.0000	96.9	3.2	0.3500	113.7	3.7	0.0005
**Mo**	100.0	2.5	0.8900	99.1	2.5	0.6900	109.2	2.1	0.0001	101.8	2.5	0.5690
**Mg**	98.2	1.3	0.2090	93.0	1.2	0.0000	102.1	1.4	0.1500	106.4	1.4	0.0000
**Ca**	98.4	1.7	0.3200	89.6	1.5	0.0000	104.8	1.8	0.0170	110.3	1.9	0.0000
**Cu**	96.4	2.0	0.1300	86.9	2.0	0.0000	101.1	2.1	0.6900	110.1	2.3	0.0002
**K**	97.3	0.8	0.0170	95.6	0.8	0.0000	101.2	0.9	0.2090	106.0	0.9	0.0000
**Na**	104.4	2.9	0.1870	89.9	2.5	0.0011	102.1	2.8	0.5620	112.9	3.2	0.0014
**P**	98.8	1.1	0.3960	92.3	1.1	0.0000	98.3	1.1	0.1390	111.5	1.3	0.0000

Table S2, (B), data from Table 3, concentration free amino acids, expressed as percentages

ratio [%]	HOSUT / wildtype			elevated / ambient			300N / 200N			+heat / -heat		
	Mean	SE	*P*	Mean	SE	*P*	Mean	SE	*P*	Mean	SE	*P*
**His**	74.5	7.0	0.0100	106.5	10.1	0.5870	116.2	10.4	0.2040	158.2	15.2	0.0000
**Asn**	86.1	3.1	0.0020	88.7	3.2	0.0110	105.5	3.9	0.2870	106.0	4.1	0.1750
**Ser**	98.7	3.2	0.5960	97.4	3.2	0.6190	102.6	3.3	0.5170	102.6	2.8	0.4630
**Gln**	84.6	7.6	0.0900	104.3	9.6	0.7530	104.3	9.2	0.7030	104.3	7.8	0.5540
**Arg**	99.1	3.8	0.8670	81.6	2.9	0.0000	108.5	4.2	0.0730	106.0	4.0	0.2270
**Gly**	94.8	1.8	0.0320	93.5	1.8	0.0070	106.0	1.7	0.0120	106.5	2.0	0.0060
**Asp**	81.7	3.9	0.0020	90.5	4.4	0.0930	103.8	5.1	0.6090	104.4	5.2	0.3960
**Glu**	94.6	1.1	0.0000	97.4	1.2	0.0800	103.8	1.2	0.0160	106.5	1.3	0.0000
**Thr**	98.6	1.9	0.2560	98.6	1.9	0.7550	102.8	2.0	0.2810	102.8	2.0	0.1030
**Ala**	93.7	1.6	0.0060	99.0	1.9	0.7740	105.3	2.1	0.0420	107.5	2.1	0.0020
**GABA**	91.5	2.9	0.0190	91.5	2.9	0.1110	116.7	3.7	0.0000	104.5	3.3	0.1230
**Pro**	91.6	3.2	0.0530	94.3	3.3	0.1580	107.1	3.7	0.0950	102.0	3.6	0.5420
**Lys**	96.6	2.4	0.2010	93.2	2.3	0.0060	101.8	2.5	0.4980	100.0	2.5	0.8820
**Tyr**	95.4	2.1	0.1160	95.4	2.1	0.0580	98.4	2.2	0.6960	101.6	2.3	0.3010
**Val**	96.6	2.4	0.2840	95.8	2.3	0.1840	108.1	2.7	0.0070	107.2	2.6	0.0120
**Ile**	97.7	3.3	0.5140	97.7	3.3	0.6160	107.3	3.6	0.1020	109.8	3.6	0.2260
**Leu**	98.2	2.5	0.3910	100.0	24.8	0.8970	103.6	2.6	0.1450	107.3	2.7	0.0070
**Phe**	98.3	2.4	0.4000	94.9	2.3	0.1050	103.6	2.6	0.1860	107.3	2.7	0.0090
**Trp**	105.0	2.5	0.0540	87.9	2.1	0.0000	101.7	2.4	0.7040	125.1	3.0	0.0000

Table S2, (C), data from Table 4, concentration storage protein classes, expressed as percentages

ratio [%]	HOSUT / wildtype			elevated / ambient			300N / 200N			+heat / -heat		
	Mean	SE	*P*	Mean	SE	*P*	Mean	SE	*P*	Mean	SE	*P*
**Crude protein**	96.1	1.4	***0.0320***	88.8	1.3	***0.0000***	107.3	1.5	***0.0010***	114.2	1.6	***0.0000***
**Extrac. prot**	96.0	1.5	***0.0360***	88.9	1.4	***0.0000***	110.2	1.7	***0.0000***	115.3	1.8	***0.0000***
**Alb/Glo**	101.9	1.9	*0.3890*	95.6	1.8	*0.0600*	103.4	1.9	*0.1430*	112.2	2.1	***0.0000***
**Gluten**	93.6	1.6	***0.0060***	86.3	1.5	***0.0000***	113.0	2.0	***0.0000***	116.7	2.1	***0.0000***
**Gliadins**	93.5	1.9	***0.0120***	85.8	1.7	***0.0000***	113.1	2.2	***0.0000***	118.3	2.3	***0.0000***
**α-gliadins**	94.0	1.7	***0.0110***	86.8	1.6	***0.0000***	112.9	2.0	***0.0000***	116.6	2.2	***0.0000***
**γ-gliadins**	94.3	2.2	*0.0500*	84.7	2.0	***0.0000***	113.4	2.6	***0.0010***	117.7	2.7	***0.0000***
**ω1,2-gliadins**	91.1	2.6	***0.0130***	86.5	2.5	***0.0010***	112.8	3.1	***0.0030***	125.5	3.4	***0.0000***
**ω5-gliadins**	90.1	2.3	***0.0060***	83.4	2.1	***0.0000***	114.4	2.9	***0.0010***	119.3	3.2	***0.0000***
**ωb-gliadins**	91.1	5.2	*0.1750*	87.7	4.9	*0.0770*	137.8	9.5	***0.0010***	101.9	6.1	*0.7820*
**Glutenin**	93.9	2.4	*0.0540*	87.4	2.2	***0.0010***	112.8	3.1	***0.0020***	112.7	3.1	***0.0020***
**HMW glutenins**	94.2	2.1	***0.0380***	90.7	2.0	***0.0020***	111.3	2.3	***0.0010***	116.8	2.6	***0.0000***
**LMW glutenins**	93.9	2.5	*0.0600*	86.1	2.3	***0.0010***	112.4	3.1	***0.0030***	111.5	3.1	***0.0040***

Fig. S1

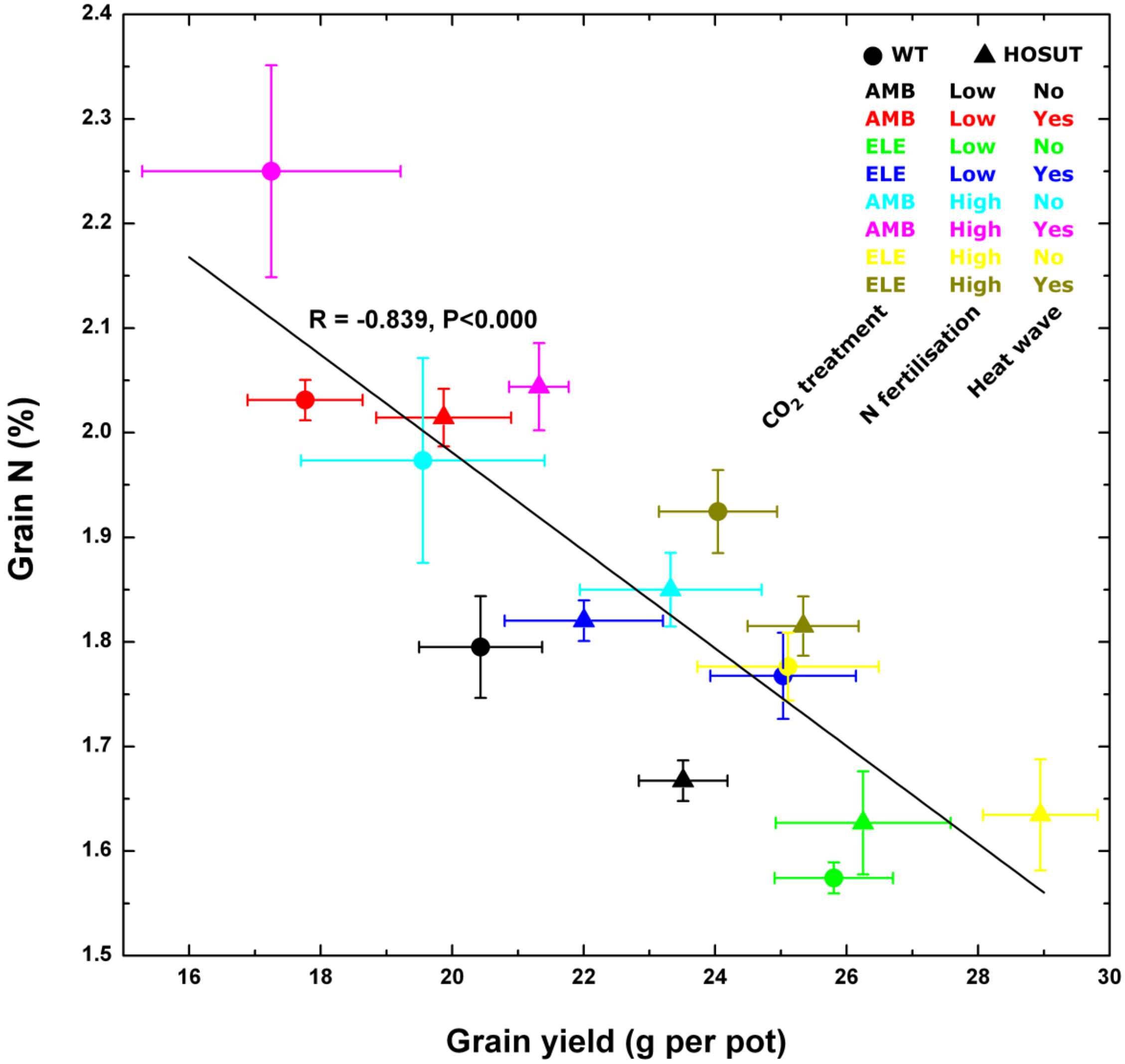

Fig. S1, Correlation between grain yield and grain quality, expressed as percentage of grain N, data are means for all 16 treatment combinations, note the significant negative association between grain yield and percentage of grain N (R = -0.844, P<0.0000). Generally all the treatments, which include the heat wave show increased percentages of grain N. However, this is not surprising because the heat wave strongly decreases grain starch content, which leads to a relative increase in grain N.

.
